# Spinning the lens on physician power: narratives of humanism and healing

**DOI:** 10.1007/s40037-019-00537-4

**Published:** 2019-09-27

**Authors:** Mercedes Chan, Laura Nimmon

**Affiliations:** 1 Department of Paediatrics, Division of Paediatric Rheumatology, University of British Columbia, Vancouver, BC Canada; 2 BC Children’s Hospital, Vancouver, BC Canada; 3 Centre for Health Education Scholarship (CHES), Faculty of Medicine, University of British Columbia, Vancouver, BC Canada; 4 Department of Occupational Science and Occupational Therapy, Faculty of Medicine, University of British Columbia, Vancouver, BC Canada

**Keywords:** Power, Humanism, Reflective practice

## Abstract

Divisive, disabling and dangerous power has featured heavily in health professions literature, social media and medical education. Negative accounts of the wielding of power have discoloured the lens through which the public sees medicine and distorted the view of a profession long associated with healing, humanism and heart. What has been buried in the midst of this discourse are positive accounts of power where the yielding of power is encouraging, empathetic and empowering. This article offers three personal vignettes illustrating the ability of power to positively affect lives in the practice of medicine, for patients and doctors alike. More of these stories are needed to uplift and rebalance the conversation on physician power and how it can be used for good. It is necessary to provide a narrative framework of what it looks like to be a healer and a humanistic doctor to satisfy the general public through a commitment to cultivate multidimensional future healthcare providers.

The medical profession is under scrutiny for how power and hierarchy function and their impact on student well-being and patient experiences [1, 2]. The exchange of power is shaped by both structural and cultural practices. For example, structural practices that foster power dynamics can be institutionally driven, as learners are dependent on positive assessments from supervisors for future job placements [2, 3]. Cultural practices can also manifest through physicians’ beliefs, values and practices about their role as ‘experts’ who share knowledge with patients, setting up a hierarchical power dynamic between physicians and patients [4, 5]. With the literature similarly mirroring the deleterious influence of medical hierarchy on student learning [6] and patient care [7], we are at risk of omitting positive narratives around physician power that can be used to exemplify physicians’ capacities for healing and empowerment.

Weaving stories of caring interactions into the lexicon of medical education can further instill healing and humanism in physician identity [8], while honouring public expectations that physicians encompass more than biomedical expertise [9]. In this paper, a paediatric rheumatologist (M.C.), shares three anecdotal stories that describe different instances of respectful yielding of physician power. M.C. and L.N., a social scientist, then offer reflections of ways that positive power can foster healing and build relationships with students and patients. All individuals whose stories are presented in this article gave their approval.

## Power to empower and engender hope

‘*I want this for you’. I will never forget these five words my colleague spoke to her patient as I watched from beside her desk that hot February morning in Cape Town. A black South African teenager from the township of Khayelitsha beamed beside me. ‘There are scholarships available for star cricket players like you. You could go to university on a scholarship! Please talk to your coach—I want this for you’. She said it again, with passion and insistence. It was this authoritative voice that caused this young man’s mother to look at my colleague with an unbelieving blank stare, taken aback that someone could imagine such a future for her son. Thirty years ago, during apartheid, this life path would have been impossible—perhaps even illegal*.

*Speaking with my colleague that day, I remarked how amazed I was that for every patient she saw, she took the opportunity to build them up, look out for their immediate well-being, and prompt them to think about their desired future—and what she could do to move them along in the right direction. I had travelled to South Africa as a visiting clinician educator; and while I learned about healthcare in Africa and the burdens of endemic disease such as HIV on rheumatology care, I came away convinced of something greater: my words were powerful—and my time with my patients can introduce meaning and hope in their lives*.

## Power to speak the truth

*As I came towards the end of my medical training, I was faced with the enviable dilemma of choosing between two jobs. Endless pros-and-cons lists, phone calls, and an anguish I had never experienced before consumed me for weeks. I was torn between the comfort and familiarity of one choice versus the excitement and opportunity of another. Both were appealing*.

*Despite overwhelming support to stay at the familiar setting, I am thankful for two physician mentors who provided their honest opinions on my offers and were quick to point out the flaws in this popular choice. I have immense respect for one mentor in particular who was critical of the popular choice despite being affiliated with that same institution. She would later prove instrumental in helping me negotiate my first job contract. The other spoke the wise words, ‘Your first job is not your last’, a phrase I now repeat to my own trainees who hold that fatalistic view that their first job out of training will also be their last.*

*I moved away from home to the shock and disappointment of many—except myself. I moved to a smaller community with a cold climate, leaps and bounds out of my comfort zone, but had four incredible years of professional and personal growth before moving again when an opportunity arose. With each move, I was in touch with my mentors who have always had my best interests at heart, and I will always appreciate that. They taught me the power to speak my truth and the power mentorship plays when it is altruistic and caring*.

## Power—the great equaliser

*My friend confided in me: ‘There were moments when our son was in the hospital and you could see in the doctors’ faces that they didn’t really know what was going on. My husband and I were really scared’. Her son had been admitted to hospital with a severe respiratory illness requiring intensive care. Both she and her husband thought they were going to lose him.*

*I told her, ‘You know, as physicians, when our patients are really sick, we get scared, too. I had a patient in the intensive care unit last week, and I was so scared that she was going to die because she was so sick. She was only 2 years old’*.

‘*You know, hearing you say that makes me feel so much better’, she responded*.

*It was an unexpected exchange at a backyard barbecue, yet one that was so instructive for me. People think that I wear an imaginary mask that symbolises my power as a physician; and when I take it off, there is a perception that we are equals. The ironic thing about this mask is that it is painted on me by society, by friends, by family, even; but it is up to me to take it off. As much as the mask affords me power, it disempowers me by concealing my own vulnerabilities and humanity. Even more important is to recognise the times when someone just wants to see the real you—unmasked. They want to know that you are human, that you identify with their pain, their grief, their joy, their experience. As physicians, I do believe we have the great privilege, as well as hold an awesome power, to be for our patients what they see as a fundamental part of being a doctor—simply human.*

## Broadening concepts of power

The first story set in South Africa is an example of how words that exercise physician power can galvanise action and empower people. Power can be used by physicians to unveil opportunities for hope and transformation in their patient populations. There is much discussion of ways patients and populations can thrive through physicians’ advocacy work. However, advocacy is more than speaking on behalf of patients and helping them access resources, as is depicted in the Royal College of Physicians and Surgeons of Canada competency framework [10]. Spoken words, themselves, can enable the imagining of multiple futures. This, for many patients, may seem limited, especially when faced with chronic or severe health issues, or for those constrained by cultural, political, economic or institutional obstacles. Spinning our lens on power, important questions to ask are, ‘What is the role of discovering patients’ hopes, goals and dreams in facilitating their journey of healing? How can physicians tap into their institutionally legitimised power to enable such discovery?’ The first and second vignettes are examples of how physicians and mentors can shape the course of patients’ and trainees’ lives. The words physicians choose can critically structure the complex ways that meaning is created through those words. In the third narrative, M.C. showed her vulnerability and humanity by describing to a friend that she is not always infallible, certain and unaffected when providing care.

Power comes into being through relationships and is present in all life relationships (e.g. marriages, friendships, families); we are never free from power. Power can be used to control, to dominate, to challenge or to enable. Seeing power as a relational concept does not deny physician authority. Rather, it challenges outdated conceptions of power as top-down, stable and determined. Social scientists have historically criticised the consequences of the hierarchical nature of our healthcare system and revealed the nature of physician power in ideology, authority and organisation [11–13]. This work, while important, has unfortunately also fostered a noticeable gap around the enabling and positive aspects of power. We are missing opportunities for conversations where physicians can use their power to invest in the lives of their patients and students.

## What next?

Physicians cannot—and should not—underestimate their cultural and symbolic power [4]. Yielding physician power mindfully can validate patients’ and team members’ sense of trust and identity, while encouraging innovative professional development and nurturing physicians’ own sense of purpose. Narratives are important tools to foster conversation about the dynamic nature of physician power. Examples that can be shared are instances of praising positive, yet subtle, professional behaviours, such as when trainees refrain from using their mobile phones during patient meetings; or stories of congratulating a teenage patient who has just been accepted into university—and the visceral experience of seeing them beam with pride! Stories of physician power can also centre on examples of empowering others by giving them permission to say ‘no’ in a culture where patients want to please their doctors and learners are often pressured to say ‘yes’ to figures of authority [14]. Moreover, integrating modalities such as trainees’ documentation of observations and reflections of role models’ positive interactions with patients and team members can generate curricular change and refresh disheartened views of physician power. Perhaps most importantly, these types of exercises can build conceptual structures of humanism and healing into the consciousness of trainees.

## Conclusion

We currently have a limited and unsophisticated understanding of physician power and how it can positively affect a consultation or an interaction with a trainee. Stories are powerful tools that can broaden conceptualisations of power in medical education and ultimately yield transformation in healthcare. In medical culture where the hidden curriculum engenders hierarchy and dominance [15, 16], positive stories of physician engagement, compassion and humanity are needed more than ever. Spinning the lens on power through rhetorical education strategies such as narratives, we may be better positioned to understand how physicians can use their words and actions meaningfully to inspire, invest in and identify with others; and by doing so, re-ignite the public’s faith in the physician as healer.
